# Diagnosis, preoperative evaluation, classification and total hip arthroplasty in patients with long-term unreduced hip joint dislocation, secondary osteoarthritis and pseudoarthrosis

**DOI:** 10.1186/s12891-020-03678-4

**Published:** 2020-10-08

**Authors:** Bo Liu, Zhaoke Wu, Zhikun Zhuang, Sikai Liu, Huijie Li, Yongtai Han

**Affiliations:** 1 Department of Osteonecrosis and Hip Surgery, the Third Hospital of Hebei Medical University, No.139 Ziqiang Road, Shijiazhuang, Hebei Province P.R. China; 2 Department of Orthopedic Surgery, Quanzhou Orthopedic-Traumatological Hospital, Quanzhou, Fujian P.R. China

**Keywords:** Hip, Dislocation, Arthroplasty, Bone deficiency

## Abstract

**Background:**

Neglected long-term unreduced hip joint dislocation with secondary osteoarthritis and pseudoarthrosis poses a great challenge to hip surgeons. However, as this is an uncommon injury, few studies have systematically investigated these patients.

**Methods:**

We retrospectively reviewed 16 patients from 2010 to 2017. The diagnostic values of three different types of common radiological examinations were evaluated. We evaluated the bone conditions of the original acetabulum and classified the patients into three types (four subtypes). The surgical procedures and prognosis of the patients were also investigated.

**Results:**

With the combined application of X-ray, CT scans and 3D reconstruction, 93.8% of these patients (sensitivity = 93.8%, Youden’s index = 0.93, intraclass correlation coefficient = 0.95) could be diagnosed correctly. There were 6/16 (37.5%) type A patients, 4/16 (25.0%) type B1 patients, 5/16 (31.3%) type B2 patients and 1/16 (6.3%) type C patient. For patients with type A injury, the surgical procedures for total hip arthroplasty were similar to “standard” total hip arthroplasty. For patients with type B injury, due to atrophy or partial bone deficiency of the original acetabulum, the surgical procedure for total hip arthroplasty was probably similar to those for patients with developmental dysplasia of the hip. For patients with type C injury, the situation was similar to that of revision surgery. The average Harris hip score postoperatively was 89.94 ± 5.78 points (range: 79–98 points).

**Conclusions:**

The new classification system could help surgeons estimate potential difficulties during total hip arthroplasty. The prognosis of most patients after total hip arthroplasty is expected to be excellent or good.

## Introduction

Traumatic fracture-dislocation of the hip joint is considered a severe injury that is commonly caused by high-violence etiologies, such as traffic accidents or falls from a height [1–3]. Generally, when traumatic fracture-dislocation of the hip joint is identified on the patient, an emergency reduction should be performed to restore the normal anatomical relationship between the femoral head and acetabulum [3]. If this fails, surgical interventions such as open reduction or periacetabular/proximal femoral osteotomy (if the hip joint remains unstable after initial treatment) may be performed to help relieve pain and promote a functional outcome [2, 4, 5].

However, the consequences if the femoral head is not reduced after injury remain unclear. It had already been reported by Pai et al. that patients with hip dislocation might be neglected in developing countries [6]. In other cases, the patient might be correctly diagnosed but cannot be treated for socioeconomic reasons [6]. Therefore, the answers regarding how to diagnose and how to treat these patients with long-term unreduced hip dislocation still have clinical usefulness. There are some reports related to patients with short-term or long-term unreduced hip joint dislocation [7–15]. The first one from Michelmore [13] in 1889 involved a 10-year-old female who was diagnosed with old unreduced dislocation of hip joint. Considering the number of years that had elapsed since the accident and the changes that must have taken place in the acetabular cavity, no attempt of reduction was made in that study. Another important point to note is that with increasing unreduced dislocation time, secondary osteoarthritis could be identified [6]. In some patients, more pathological findings could be identified. Pai [6] reported 26 patients with unreduced posterior hip dislocations. In four of them (all neglected more than 1 year), the formation of a “pseudoacetabulum” could be identified. In summary, these patients had the following characteristics: (1) the femoral head was located in the posterior-superior direction relative to its original position; (2) a pseudoacetabulum was formed around the dislocated femoral head; (3) pathoanatomical changes were present in the original acetabulum; (4) secondary osteoarthritis; and (4) limb shortening.

**Fig. 1:**
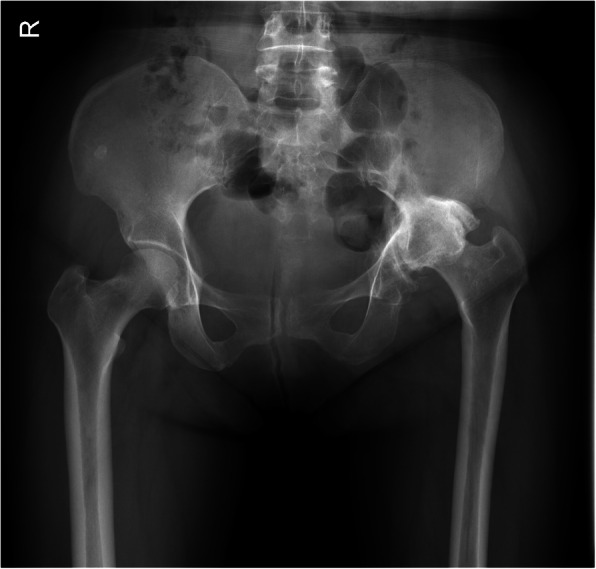
A 52-year-old female patient had been referred because of left hip pain for the past year. She had reported a previous “fall from a height” 3 years ago, which caused an “acetabular fracture”. The patient received conservative treatment. The radiograph showed “osteoarthritis” of her left hip joint, a condition that is apt to be misdiagnosed

Hip pain and dysfunction identified in a patient might be easily misdiagnosed as common osteoarthritis, especially in the presence of radiological findings similar to typical osteoarthritis (Fig. 1). However, we have not found any studies about the diagnosed value of different radiological examinations for patients with long-term unreduced hip joint dislocation with secondary osteoarthritis and pseudoacetabulum formation, as this is an uncommon injury and few centers collect sufficient cases to be able to appraise their methods of treatment realistically. Total hip arthroplasty using artificial joint prosthesis replacement might be considered the optimal treatment option because patients could be expected to be pain-free and have a satisfactory functional outcome postoperatively [8, 16]. However, the unique characteristics of surgical procedures in these patients are also rarely reported [7, 15, 17]. In particular, long-term dislocation and pseudoacetabulum formation may have an effect on the surgical approach and acetabular component implantation. In this study, 16 patients with long-term (more than 3 years) unreduced hip joint dislocation were retrospectively reviewed. All patients had secondary osteoarthritis, significant clinical symptoms and pseudoacetabulum formation. The patients were all treated with total hip arthroplasty with artificial joint replacement. The aim of our study was to evaluate the diagnostic value of different radiological examinations, to classify the patients and to provide surgical techniques that, in our opinion, would be helpful for surgeons to manage such patients with long-term unreduced hip joint dislocation, secondary osteoarthritis and pseudoarthrosis.

## Patients and methods

### Study population

We retrospectively reviewed 16 patients who were treated in our hospital for long-term unreduced hip joint dislocation, secondary osteoarthritis and pseudoarthrosis from 2010 to 2017. Note that in this study, “pseudoarthrosis” means the false joint formation between the dislocated femoral head and the neo-formed osteophyte (pseudoacetabulum), rather than the result of fracture-nonunion. This unique characteristic helps distinguish these unique patients from common patients with fracture-nonunion or an old fracture. Patients were excluded if they had a certain history of developmental dysplasia of the hip or if the time period between hip dislocation and surgery was less than 1 year. The general information, history, etiology, and comorbidity injuries of patients were identified based on either patient reports or medical records. The study was approved by the Institutional Review Board of the Third Hospital of Hebei Medical University and was conducted in accordance with the Declaration of Helsinki and regulations of the Health Insurance Portability and Accountability Act (HIPAA). As this was a retrospective study and all patient information was deidentified before analysis, informed consent was not required except for patients whose radiological images would be published.

### Evaluation of diagnostic methods

Because of the easily confused radiological diagnosis, our purpose was to evaluate the diagnostic value of three different kinds of common radiological examinations: (1) standard anterior-posterior view and lateral view X-ray examinations, (2) computed tomography (CT) with multiplane reconstruction (axial position and coronal position), and (3) computed tomography with 3D reconstruction.

Initial sample size calculations assumed that X-ray examinations were 20% sensitive for the diagnosis of these patients. When the permissible error and significance level were set to 5% and 0.05, respectively, the needed patients and controls were 246 each. However, because patients with long-term unreduced hip joint dislocation, secondary osteoarthritis and pseudoarthrosis were very rare, the sample size of patients was limited to 16 patients. For convenience of calculation, we included 16 patients and 284 controls. As such, special attention was needed with regard to the overestimated specificity due to the limited sample size. In addition, a “normal” diagnostic test notably requires the distinction between “patients” and the “healthy population”. However, in our study, patients with long-term hip joint dislocation, secondary osteoarthritis and pseudoarthrosis could be distinguished easily from the healthy population. Therefore, we did not choose a healthy population as our control. Instead, we selected patients with other hip disorders, including osteoarthritis, as our controls. Therefore, the sensitivity (as well as other indexes such as specificity, accuracy, etc.) in our study represented the ability of the diagnostic method to distinguish between “patients with long-term unreduced hip joint dislocation, secondary osteoarthritis and pseudoarthrosis” and “controls (patients with other hip disorders, such as osteoarthritis, osteonecrosis of the femoral head, etc.)” instead of a “healthy population”. Finally, a total of 284 individuals, including 177 patients with osteoarthritis, 58 patients with osteonecrosis of the femoral head, 23 patients with developmental dysplasia of the hip (DDH), 18 patients with rheumatoid arthritis, and 8 patients with old femoral neck fractures were included as controls.

A panel of 4 readers (2 orthopedic surgeons and 2 radiologists) independently evaluated each radiological examination while blinded to the clinical information and all patient identifiers. None of the 4 readers were involved in the clinical care or evaluation of the enrolled patients. Interpretations for each radiological examination (only X-ray, X-ray & CT and X-ray & CT & 3D reconstruction) for a single patient were performed on different days sequentially to avoid reader recognition or recall of findings from the other radiological examination. The order of presentation of the radiological images for different patients was randomized and differed for each reader. The diagnosis of the patient was recorded. If unanimous agreement on an individual radiological examination was not achieved by each of the 4 readers, the interpretation of the majority of readers was used as the final radiological diagnosis. In evenly distributed disagreements (2 vs 2), final interpretation was reached by a group consensus discussion. Since the aims of our study were to evaluate the diagnostic value of different kinds of radiological examinations for patients with long-term unreduced hip joint dislocation, secondary osteoarthritis and pseudoarthrosis, we did not record misdiagnoses between other hip disorders (for example, if a patient with osteonecrosis was misdiagnosed as having osteoarthritis, this individual was not regarded as a misdiagnosed individual in our study). The final hospital discharge diagnosis, which was regarded as the “gold standard” diagnosis incorporating all available histories, clinical manifestations, radiological data and findings during surgery, was made at the time of discharge by the surgeon.

Indexes, such as sensitivity, specificity, accuracy, Youden’s index and intraclass correlation coefficient, were calculated to demonstrate the diagnostic value of these three different types of radiological examinations.

### Radiological measurements and classification

**Fig. 2:**
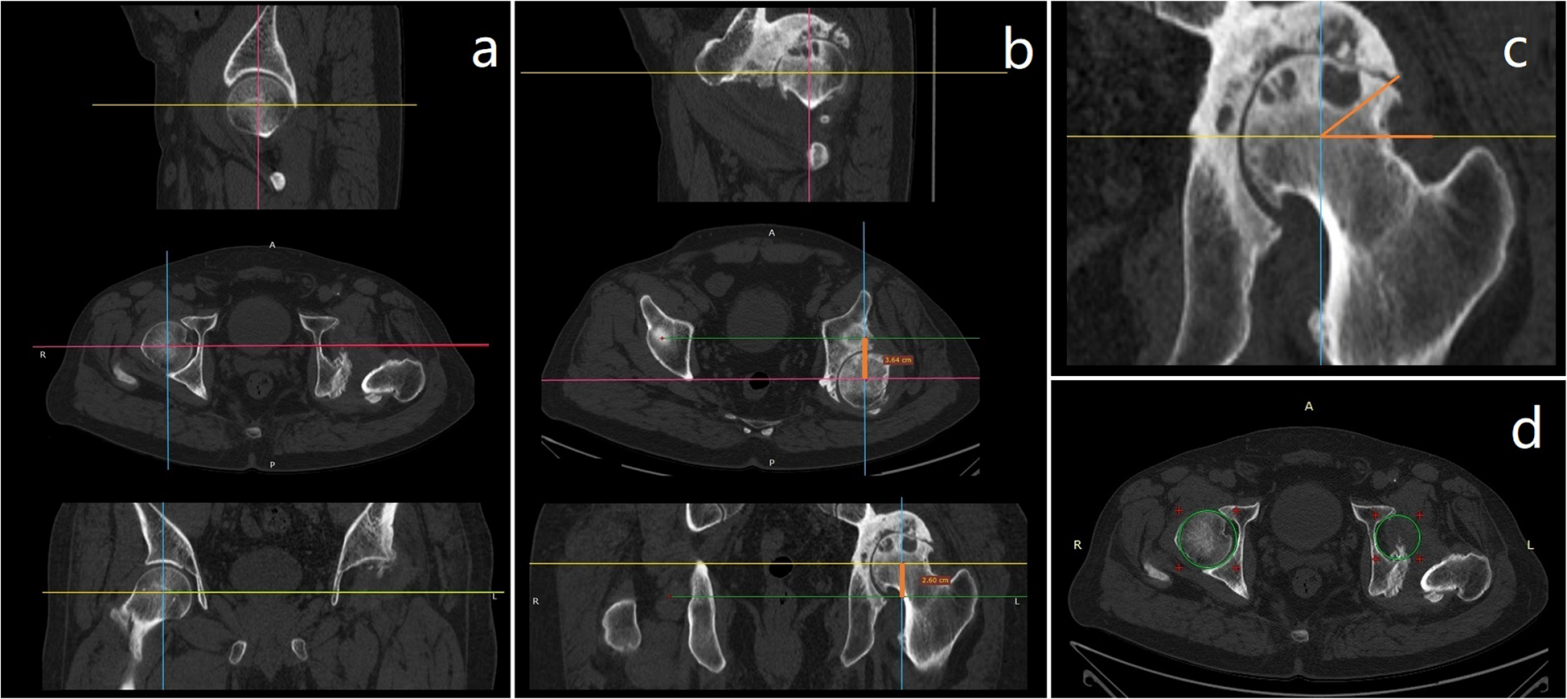
Radiological measurements of patients with long-term unreduced hip joint dislocation, secondary osteoarthritis and pseudoarthrosis. **a** On the “standard” (bilateral symmetry of the patient in every direction) multiplanar reconstruction image, the rotational center of the contralateral femoral head is identified. **b** The rotational center of the ipsilateral femoral head is identified. The upward shift distance (backward shift distance) of the rotational center is defined as the vertical distance (orange color) between the rotational center of the ipsilateral femoral head and the level of the contralateral femoral head center. **c** The coverage of the “pseudoacetabulum” is defined as the angle between the horizontal line of the rotational center of the ipsilateral femoral head and the lateral edge of the pseudoacetabulum. **d** On the standard multiplanar reconstruction image, the acetabular diameter is identified at the level of the rotational center of the contralateral femoral head

The radiological measurements of patients with long-term unreduced hip joint dislocation, secondary osteoarthritis and pseudoarthrosis were also performed to demonstrate the displacement direction of the rotational center, the coverage of pseudoacetabulum and the bony deficiency of the original acetabulum. All the radiological measurements were performed from the multiplane-reconstruction images of CT scans by an electronic ruler or protractor (RadiAnt DICOM Viewer 5.0, Medixant, Poznan, Poland). The upward shift distance (backward shift distance) of the femoral head was defined as the shortest distance between the rotational center of the ipsilateral femoral head and the level of the contralateral femoral head center (Fig. 2) from coronal images (axial images). The coverage of the pseudoacetabulum was also evaluated by the angle between the horizontal line of the rotational center of the ipsilateral femoral head and the lateral edge of the pseudoacetabulum (Fig. 2). The diameters of the ipsilateral original acetabulum and the contralateral acetabulum were also measured (Fig. 2). All the radiological measurements were completed by the same experienced orthopedic surgeon. To test the intraobserver reproducibility, this surgeon performed all the radiographic measurements in 5 randomly selected patients. The measurements were repeated after 2 weeks. The intraclass correlation coefficient was used to assess intraobserver reliability. The results showed good reliability (intraclass correlation coefficient > 0.9 in all measurements). The original acetabular bone deficiency was classified according to the Paprosky classification system.

According to the bone conditions of the original acetabulum, we classified the patients into three types (with four subtypes).

### Surgical procedure and outcome

Information on the surgical procedure was collected from the medical records. All surgeries for all patients were performed by the same group of surgeons. Preoperative templating was not routinely used for any cases. However, in some cases, computed-assisted preoperative plans or 3D printing technology were performed instead. The approaches, implanted position of the acetabular component, and selection of both the acetabular and femoral components were identified. Full weightbearing was allowed on the day after surgery (except those with bone grafting or augment).

The primary outcome was the Harris score and excellent-good rate at the last follow-up, which indicated the pain relief and functional recovery. The secondary outcome was the incidence of complications, including both early complications (such as deep vein thrombosis, early periprosthetic fractures, etc.) and late complications (such as heterotopic ossification, late prosthetic dislocation, aseptic loosen, etc.).

To make the results comparable, some patients with similar characteristics were also enrolled as the controls. Because patients with long-term unreduced hip joint dislocation, secondary osteoarthritis and pseudoarthrosis were classified into three types in this study, three other types of patients with different hip disorders were included as the controls, namely, 100 patients with osteonecrosis of the femoral head, 100 patients with dysplasia of the hip and 100 patients with aseptic loosen of the hip prosthesis. All these controls had undergone total hip arthroplasties (or revision surgery). The demographic information and follow-up time of these controls were compared with those of all 16 of the patients with long-term unreduced hip joint dislocation. The results showed good comparability (no significant differences were found regarding the demographic information and follow-up time between the patients and controls). Note that for the control group, if a patient received bilateral surgeries, he or she was considered as two independent individuals.

### Statistical analysis

Statistical analyses were performed using SPSS 19.0 statistical software for Windows (IBM, Armonk, NY) and Excel 2016 for Windows (Microsoft Corporation, Seattle, WA). Continuous variables were expressed as the mean ± standard deviation, and categorical variables were expressed as frequencies. Sensitivity, specificity, accuracy, Youden’s index and the intraclass correlation coefficient were calculated to indicate the diagnostic value of the different kinds of radiological examinations. Cochran’s Q tests were used to identify the differences in sensitivities between groups. The Mann-Whitney U test was performed to identify the differences in continuous variables between the groups. The chi-square test was performed for categorical variables. A *P* value less than 0.05 was considered to be significant.

## Results

### General information

A total of 16 patients were finally included in this study. All patients had a history of traumatic injury. There were 9 male patients and 7 female patients. The mean age was 57.44 ± 11.38 years (range from 38 years to 76 years). Among all patients, injuries occurred most commonly via traffic accidents (10/16 patients). Injuries in 4 patients occurred via falls from a height and in 2 patients occurred via crushing injuries. All of these patients reported that they had hip pain and restricted movement after the initial trauma. None of them reported surgical treatment at the time of injury. Six out of 16 patients either reported a certain history of hip joint dislocation or had a medical record at the time of injury that could confirm hip joint dislocation. In other patients, there was no certain evidence to demonstrate whether there was a hip joint dislocation at the time of injury. Comorbidity fractures of patients were also investigated. Among all 16 patients, 11 had reported or currently diagnosed comorbidity fractures of one or more sites. The most common comorbidity fractures were acetabular fractures (5/11 patients). No patient had been identified with significant avascular necrosis of the femoral head at the time of hip arthroplasty. The average time from the initial injury to total hip arthroplasty was 9.69 ± 5.21 years (range from 3 years to 23 years). All patients had serious hip pains and limited motion. The average Harris score was 48.88 ± 9.12 points (range from 35 points to 63 points). The demographic characteristics, etiologies, comorbidities and hip functional scores are shown in Table 1.

**Table 1:** Demographic and general characteristics of patients with long-term unreduced hip joint dislocation, secondary osteoarthritis and pseudoarthrosis

Case	Gender	Age (years)	Side	Etiology	Comorbidity injuries	Time from initial injury to surgery (years)	Harris score preoperatively
1	Female	56	Left	Crushing Injury	Superior pubic ramus fracture	23	55
2	Male	59	Left	Traffic accident	Patella fracture, tibiofibular fracture, iliac fracture	9	38
3	Male	62	Right	Fall from a height	Calcaneal fracture	6	46
4	Male	65	Right	Crushing Injury	–	7	59
5	Female	46	Right	Traffic accident	Acetabular fracture	9	42
6	Male	38	Left	Fall from a height	–	6	35
7	Female	71	Right	Traffic accident	Femoral shaft fracture	6	44
8	Female	59	Left	Traffic accident	–	8	63
9	Female	52	Left	Fall from a height	Acetabular fracture	3	46
10	Male	42	Right	Traffic accident	Inferior pubic ramus fracture	13	61
11	Female	39	Left	Traffic accident	Acetabular fracture	9	36
12	Male	70	Left	Traffic accident	Acetabular fracture	12	59
13	Male	63	Right	Traffic accident	–	19	55
14	Female	76	Right	Traffic accident	Acetabular fracture	5	42
15	Male	59	Left	Fall from a height	Ischial fracture	8	52
16	Male	62	Left	Traffic accident	–	12	49

### Radiological diagnosis

**Fig. 3:**
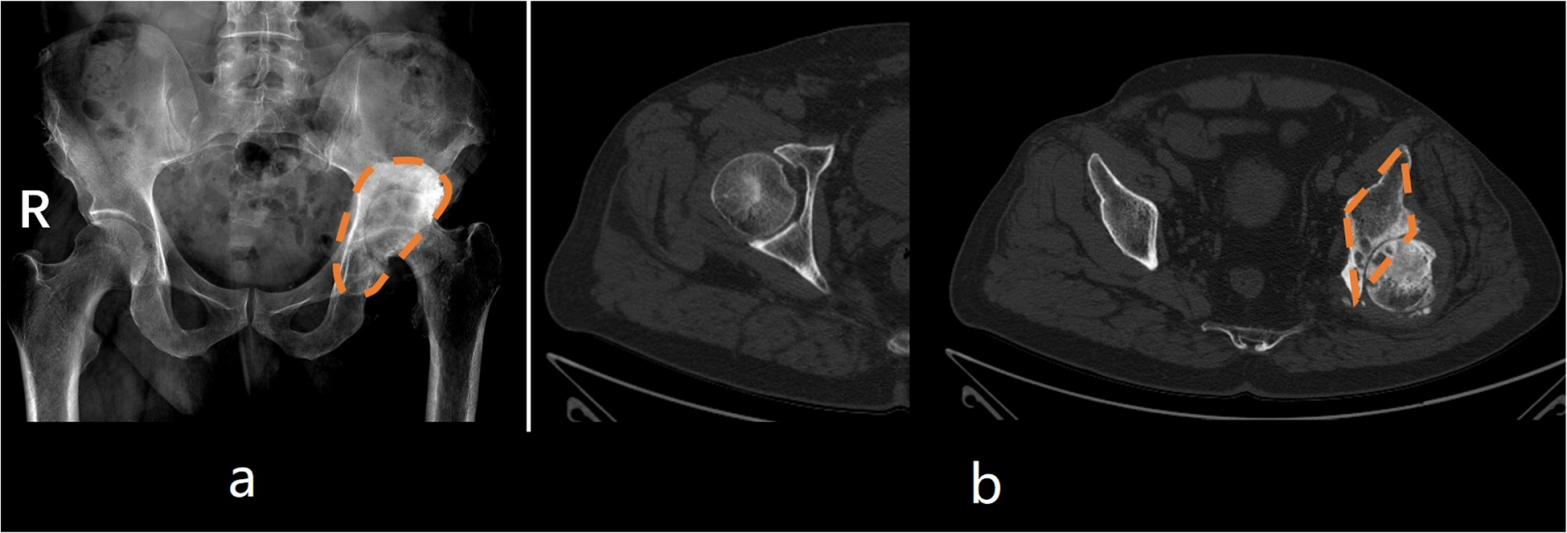
Radiological characteristics of patients with long-term unreduced hip joint dislocation, secondary osteoarthritis and pseudoarthrosis. **a** On normal anterior-posterior view X-ray examination, a semicircular high-density area could be identified in typical cases. Because the area looks like the lunar aureole (pseudoacetabulum) around the moon (femoral head), it is called the “aureole” sign. When an aureole sign is identified together with a slightly upward shift of the rotation center of the hip joint, the diagnosis of long-term unreduced hip joint dislocation, secondary osteoarthritis and pseudoarthrosis must be considered to avoid the possibility of misdiagnosis. There is no objective quantitative index to describe the aureole sign. We estimate that the aureole sign can be observed in 13/16 patients (this is based on subjective results obtained by one observer). **b**. On axial CT, when the rotation center is identified, the anterior and posterior column and the internal wall of the acetabulum resemble a large letter “I” in healthy people. However, in patients with long-term unreduced hip joint dislocation, secondary osteoarthritis and pseudoarthrosis, the ilium, dome of the original acetabulum and the internal/posterior wall of the pseudoacetabulum will form a rhombus, which is called the “rhombus” sign. This sign might be helpful in avoiding the possibility of misdiagnosis. There is no objective quantitative index to describe the rhombus sign. We estimate that the rhombus sign can be observed in 11/16 patients (this is based on subjective results obtained by one observer)

The diagnostic values of different kinds of radiological examinations are shown in Table 2. On standard X-ray examinations, only 18.8% of patients (sensitivity = 18.8%, Youden’s index = 0.18, intraclass correlation coefficient = 0.21) with long-term unreduced hip joint dislocation, secondary osteoarthritis and pseudoarthrosis could be distinguished from patients with other hip disorders. Unreduced hip joint dislocation with secondary osteoarthritis and pseudoarthrosis was most commonly misdiagnosed as an osteoarthritis (Fig. 1). Then, with the axial and coronal images of CT scans, 50.0% of this category of patients (sensitivity = 50.0%, Youden’s index = 0.49, intraclass correlation coefficient = 0.69) could be distinguished from patients with other hip disorders. On CT scans, patients with long-term unreduced hip joint dislocation, secondary osteoarthritis and pseudoarthrosis were most commonly misdiagnosed as having an acetabular retroversion deformity with osteoarthritis. Finally, with the combined application of X-ray, CT scans and 3D reconstruction, 93.8% of these patients (sensitivity = 93.8%, Youden’s index = 0.93, intraclass correlation coefficient = 0.95) could be diagnosed correctly. Compared to the sensitivity of the combined application of X-ray, CT scans and 3D reconstruction for diagnosis, the sensitivities were significantly lower when only X-ray examinations (*P* < 0.001) or X-ray with axial and coronal CT scans (*P* = 0.016) were used. The Youden’s indexes and intraclass correlation coefficients also increased gradually along with an increase in the number of combined diagnostic methods. In particular, we found two radiological signs (one on normal X-ray examinations and one on standard axial CT scans) that might help radiologists or orthopedic surgeons to distinguish potential patients with long-term unreduced hip joint dislocation, secondary osteoarthritis and pseudoarthrosis from “normal” osteoarthritis patients (Fig. 3).

**Table 2:** Diagnosis of patients with long-term unreduced hip joint dislocation, secondary osteoarthritis and pseudoarthrosis

	Only X-ray	X-ray + CT^a^	X-ray + CT^a^ + 3D reconstruction
Sensitivity (95% CI)	18.8% (0–37.9%)^b^	50.0% (25.5–74.5%)^c^	93.8% (81.9–100%)
Specificity (95% CI)	98.9% (97.8–100%)	98.9% (97.8–100%)	99.6% (99.0–100%)
Accuracy (95% CI)	94.7% (94.3–95.0%)	96.3% (95.9–96.7%)	99.3% (99.1–99.5%)
Youden’s index	0.18	0.49	0.93
Intraclass correlation coefficient	0.21	0.69	0.95

*CI* confidence interval, *CT* computed tomography

^a^Axial and coronal scanning

^b^ The sensitivity was significantly lower than that of “X-ray + CT + 3D reconstruction” (P < 0.001)

^c^ The sensitivity was significantly lower than that of “X-ray + CT + 3D reconstruction” (*P* = 0.016)

### Radiological measurements

The purpose of radiological measurement is to help surgeons understand the dislocation direction of the femoral head and the bone condition around the hip joint. As there were posterior dislocations of the hip joint in all these patients, the rotational centers had a positive upward shift and a positive backward shift in all cases. The average upward and backward shift distances of the rotational center were 27.04 ± 2.91 mm (range from 22.3 mm to 30.6 mm) and 27.75 ± 5.42 mm (range from 15.1 mm to 36.4 mm), respectively. The average coverage of pseudoacetabulum was 43.79 ± 8.89 degrees (range from 23.5 degrees to 55.8 degrees). The original acetabulum was found to be atrophied following the dislocation of the femoral head in some cases. The average diameters of the contralateral acetabulum (D) and ipsilateral acetabulum (d) were 49.04 ± 3.82 mm (range from 39.8 mm to 55.2 mm) and 44.05 ± 6.87 mm (range from 33.5 mm to 56.3 mm), respectively. According to the Paprosky classification system, there were 3 patients with acetabular bone deficiency of type I, 1 patient with acetabular bone deficiency of type IIA and 1 patient with acetabular bone deficiency of type IIB. The results of the radiological measurements are shown in Table 3.

**Table 3:** Radiological measurements of patients with long-term unreduced hip joint dislocation, secondary osteoarthritis and pseudoarthrosis

Case	Upward shift distance of the rotational center (mm)	Backward shift distance of the rotational center (mm)	Coverage of the pseudoacetabulum (°)	Contralateral acetabular diameter (D, mm)	Original acetabular diameter (d, mm)	Original acetabular bone deficiency
1	30.5	32.4	43.1	44.6	33.5	–
2	26.0	36.4	37.2	47.6	38.8	–
3	29.4	30.6	38.1	51.6	52.1	Paprosky I
4	29.9	35.8	53.6	52.0	51.8	–
5	25.8	24.8	34.5	39.8	38.5	Paprosky I
6	30.6	28.6	38.5	55.2	56.3	–
7	23.6	29.2	50.2	48.6	49.1	–
8	26.2	23.8	47.9	50.1	39.6	–
9	27.6	15.1	23.5	44.5	45.0	Paprosky IIB
10	22.8	26.5	55.8	51.8	40.6	–
11	30.6	28.7	52.5	50.8	41.6	–
12	22.3	29.6	39.0	53.5	55.2	–
13	27.5	22.8	53.1	49.6	38.2	–
14	26.5	23.0	52.3	46.8	42.1	Paprosky IIA
15	23.6	28.9	42.8	49.5	38.0	–
16	29.8	25.8	38.6	48.6	44.4	Paprosky I

### Classification

**Fig. 4:**
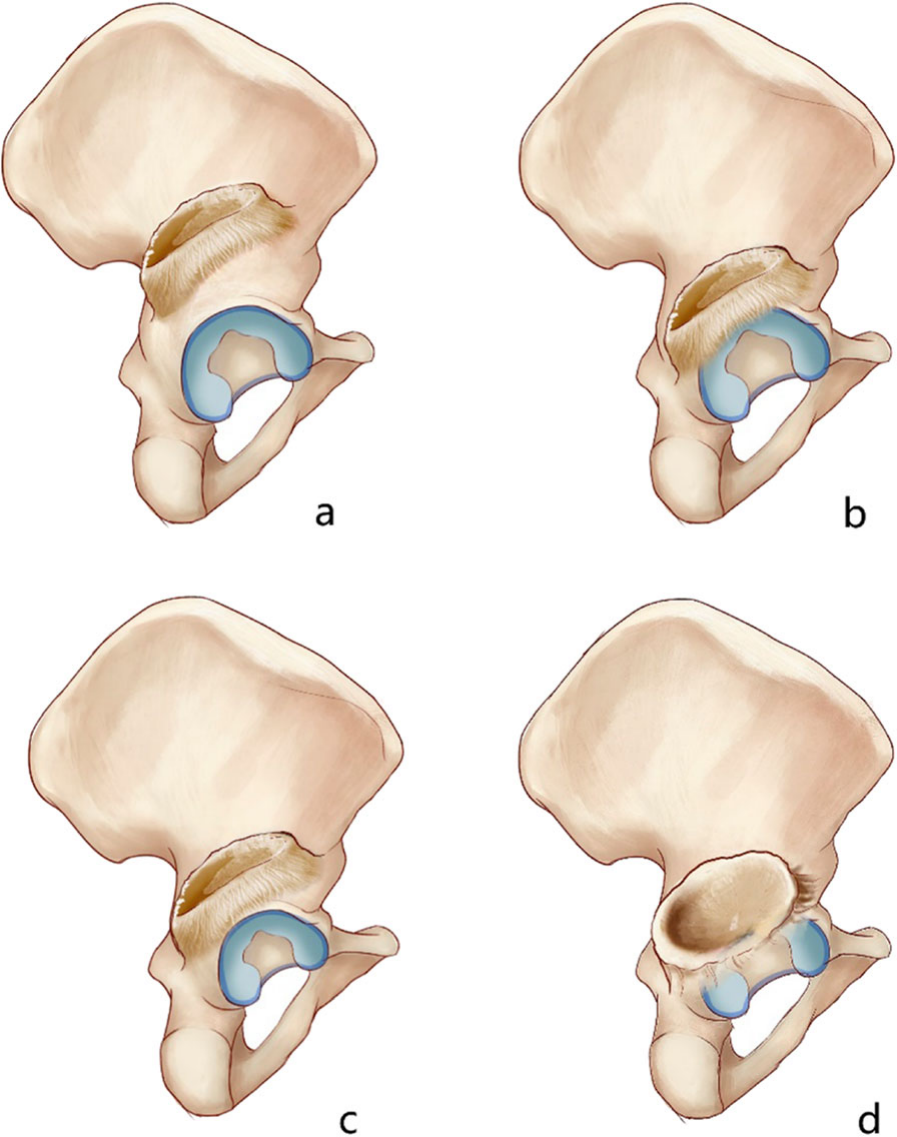
Diagram for the classification system of patients with long-term unreduced hip joint dislocation, secondary osteoarthritis and pseudoarthrosis. **a** Type A, the original acetabulum remains nearly normal in size and has morphological features that are not affected by the inferior-anterior wall of the pseudoacetabulum. **b** Type B1, the original acetabulum remains nearly normal in size, but the superior-posterior wall is invaded by the inferior-anterior wall of the pseudoacetabulum, which leads to bone deficiency of the posterior wall of the original acetabulum. **c** Type B2, the original acetabulum is atrophied and is not affected by the inferior-anterior wall of the pseudoacetabulum. **d** Type C, almost the whole original acetabulum is invaded by the pseudoacetabulum, which leaves a severe bone deficiency, or the original acetabulum cannot be identified

According to the bone conditions of the original acetabulum, we propose a new classification system for these patients, which could help surgeons estimate potential difficulties during total hip arthroplasty (Table 4 and Fig. 4). Patients who were classified with type A were characterized to have a “nearly normal” original acetabulum, which means that the diameter of the ipsilateral acetabulum was nearly equal to the diameter of the contralateral acetabulum (D/d > 0.8), with no significant bone deficiency of the affected acetabulum. There were two subtypes of patients with type B disease. In patients with type B1, the original acetabular diameter was similar to that in patients with type A, but bone deficiency could be identified in the dome area or posterior wall of the acetabulum. In patients with type B2, no significant bone deficiency was identified, but the ipsilateral original acetabular diameter was significantly lower than the diameter of the contralateral acetabulum (D/d ≤ 0.8). Finally, patients of type C would be expected to have severe bone deficiency around the original acetabulum, or the original acetabulum could not be identified in radiological examinations or during the surgery. There were 6/16 (37.5%) patients with type A, 4/16 (25.0%) patients with type B1, 5/16 (31.3%) patients with type B2 and 1/16 (6.3%) patient with type C.

**Table 4:** Classification system for patients with long-term unreduced hip joint dislocation, secondary osteoarthritis and pseudoarthrosis

Classification	Characteristic	Morphological characteristics of the original acetabulum	Original acetabular bone deficiency	Morphological characteristics of the proximal femur	Proportion (%)
Type A	Normal original acetabulum diameter, without bone deficiency	Nearly normal (D/d > 0.8)	Integral	Nearly normal	6/16 (37.5)
Type B1	Normal original acetabulum diameter, partial bone deficiency	Nearly normal (D/d > 0.8)	Dome or posterior wall deficiency		4/16 (25.0)
Type B2	Atrophied original acetabulum, without bone deficiency	Atrophied (D/d ≤ 0.8)	Integral		5/16 (31.3)
Type C	Severe bone deficiency or unidentified original acetabulum	Normal, atrophied or unidentified	Large dome and posterior wall deficiency, acetabular column deficiency		1/16 (6.3)

D, contralateral acetabular diameter; d, original acetabular diameter

### Surgical treatment and prognosis

**Fig. 5:**
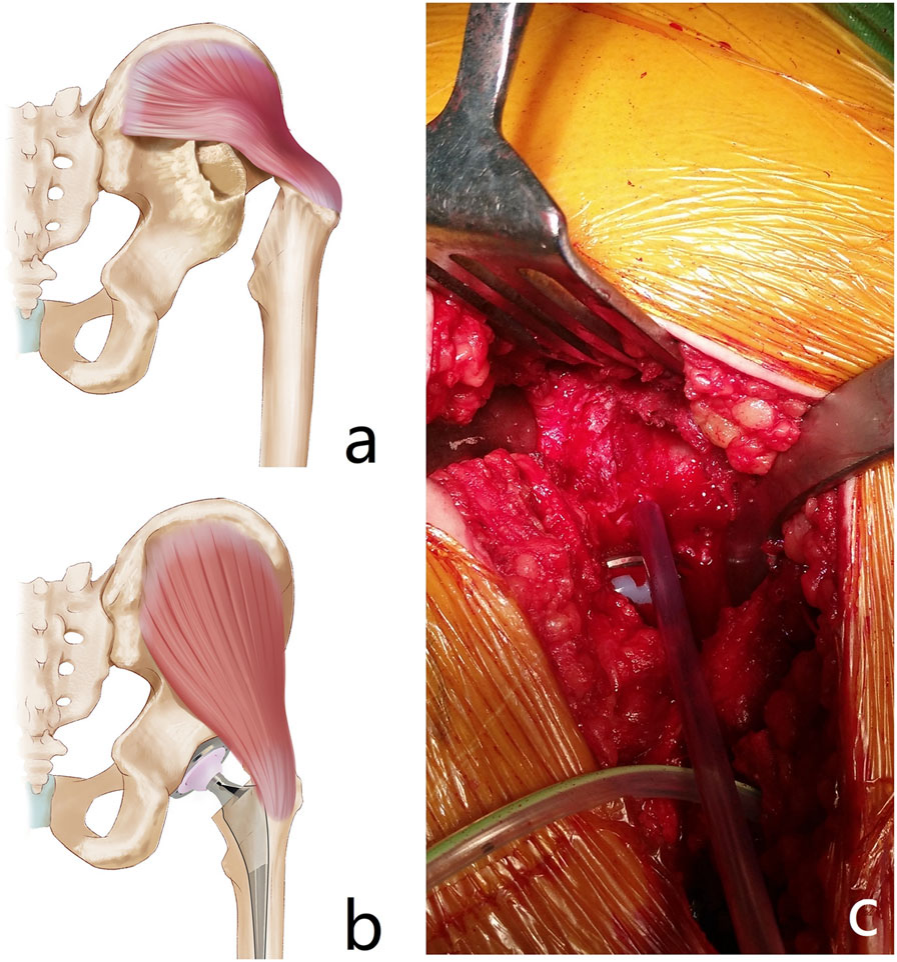
Gluteus medius (and gluteus minimus) originates from the tip of the greater trochanter and terminates on the posterior-superior border (posterior-lateral surface) of the ilium. **a** In patients with long-term unreduced hip joint dislocation, secondary osteoarthritis and pseudoarthrosis, the gluteus medius and gluteus minimus are pushed away from the surface of the ilium by the pseudoacetabular bone osteophyte, which increases muscle tension and causes failure to reduce the joint prosthesis. **b** When the pseudoacetabular bone osteophyte is removed, the muscle tension decreases dramatically, and the joint prosthesis can be easily reduced. **c** This intraoperative photograph shows the osteophyte and the gluteus medius

All patients received total hip arthroplasty. Thirteen out of 16 patients received surgery via the posterior approach, and 3/16 patients received surgery via the anterior approach. All the acetabular components (e.g., Pinnacle Gription from DePuy, Trabeculae Oriented Pattern cup from Link, Trabecular Metal Cup from Zimmer, etc.) were placed on the original acetabular socket without the combined application of a restrictive liner. In 6/16 patients with type A, standard acetabular component was implanted without bone grafting or augmentation. In 4/16 patients with type B1, structural bone grafting (or augmentation) was performed to fill the bony deficiency of the original acetabulum. In 5/16 patients with type B2, small-size acetabular component was used. In two of these patients, because the cancellous bone of the original acetabulum was atrophied, nonstructural bone grafting was performed. In one patient with type C, multiple augmentations were performed to help fill the bone deficiency. Except for one patient was implanted with a modular femoral component (S-rom from DePuy) for femoral side surgery, all other patients were implanted with “standard” femoral components (e.g., Tri-Lock from DePuy, Link Classic Uncemented from Link, M/L Taper from Zimmer, etc.). If there were difficulties in the process of hip joint reduction, removing the pseudoacetabular bone osteophyte was likely to be helpful (Fig. 5).

The average follow-up time of patients was 4.07 ± 1.60 years (range from 2.2 years to 8.5 years). All prostheses survived at the last follow-up. The average Harris hip score was 89.94 ± 5.78 points (range from 79 points to 98 points). Except for one patient who had a fair outcome, all other patients achieved excellent-good results. Superficial surgical site infection was identified in one patient who had a history of diabetes and was cured after debridement and wound dressing (together with antibiotic application). Deep vein thrombosis was identified in two patients and was cured by continuous use of anticoagulants. Mild heterotopic ossification (Brooker grade 2) was identified in two patients, with no special treatment being offered. The surgical treatments and prognosis for patients with long-term unreduced hip joint dislocation, secondary osteoarthritis and pseudoarthrosis are shown in Table 5. A comparison between the patients and controls showed no significant differences with respect to surgical technique, Harris score at last follow-up or incidence of complications (Table 6).

**Table 5:** Surgical treatments and prognosis for patients with long-term unreduced hip joint dislocation, secondary osteoarthritis and pseudoarthrosis

Case	Type	Approach	Acetabular Component	Femoral Component	Bone Grafting	Harris Score	Complications	Follow-up time (years)
1	B2	Posterior	Pinnacle Gription (DePuy)	Tri-Lock (DePuy)	–	88	DVT	2.2
2	A	Posterior	R3 Think (Smith-Nephew)	SL-MIA (Smith-Nephew)	–	92	–	3.5
3	B1	Posterior	Pinnacle Gription (DePuy)	S-Rom (DePuy)	Structural	90	DVT	4.8
4	A	Anterior	Combi Cup (LINK)	L.C.U. (LINK)	–	96	–	3.0
5	B1	Posterior	Trabecular Metal Cup (Zimmer)	M/L Taper (Zimmer)	Augment	82	–	2.9
6	A	Posterior	Combi Cup (LINK)	Ribbed (LINK)	–	89	–	5.6
7	A	Posterior	R3 Think (Smith-Nephew)	SL-MIA (Smith-Nephew)	–	79	HO	8.5
8	B2	Anterior	T.O.P. (LINK)	L.C.U. (LINK)	Nonstructural	95	–	3.1
9	C	Posterior	Trabecular Metal Cup (Zimmer)	M/L Taper (Zimmer)	Augment	82	–	2.6
10	B2	Posterior	T.O.P. (LINK)	L.C.U. (LINK)	–	92	SSI	5.0
11	A	Anterior	Duraloc (DePuy)	Tri-Lock (DePuy)	–	95	–	2.6
12	A	Posterior	T.O.P. (LINK)	L.C.U. (LINK)	–	98	–	3.2
13	B2	Posterior	Pinnacle Gription (DePuy)	Tri-Lock (DePuy)	Nonstructural	98	–	4.5
14	B1	Posterior	Duraloc (DePuy)	Corail (DePuy)	Augment	90	HO	4.2
15	B2	Posterior	T.O.P. (LINK)	L.C.U. (LINK)	–	87	–	3.8
16	B1	Posterior	Trabecular Metal Cup (Zimmer)	M/L Taper (Zimmer)	Structural	86	–	5.6

DVT, deep vein thrombosis; HO, heterotopic ossification; SSI, surgical site infection; L.C.U., link classic uncemented; T.O.P., trabeculae oriented pattern

**Table 6:** Comparison of surgical techniques, function outcome and complications between patients with long-term unreduced hip joint dislocation, secondary osteoarthritis and pseudoarthrosis and controls

Characteristics		Type A			Type B			Type C		
		Patients (*n* = 6)	Controls (*n* = 100)	P	Patients (*n* = 9)	Controls (*n* = 100)	P	Patients (*n* = 1)	Controls (*n* = 100)	P
Surgical technique										
Approach (n)	Anterior	2	15	0.235^b^	1	7	0.651^b^	0	0	–
	Posterior	4	85		8	93		1	100	
Shell diameter (mm)		49.67 ± 2.34	50.44 ± 4.02	0.648^a^	44.00 ± 5.10	45.46 ± 4.21	0.334^a^	50.00	53.36 ± 4.88	–
Bone grafting^c^ (n)	N/A	6	92	0.471^b^	3	67	0.108^b^	0	23	–
	Non-Structural	0	8		2	8		0	45	
	Structural^d^	0	0		4	25		1	32	
Harris Score		91.50 ± 6.89	92.87 ± 4.82	0.794^a^	89.78 ± 4.82	88.97 ± 6.44	0.578^a^	82.00	81.24 ± 10.89	–
Complication (n)	N/A	5	85	0.566^b^	5	72	0.286^b^	1	70	–
	DVT	0	11		2	15		0	16	
	Heterotopic ossification	1	3		1	3		0	7	
	SSI	0	2		1	2		0	5	
	Dislocation	0	2		0	8		0	5	
	Periprosthetic fracture	0	1		0	7		0	4	
	Aseptic loosen	0	0		0	0		0	1	

Note: Because more than one type of complication could be identified in a single patient, the overall count of complications may be more than the number of patients

*DVT* deep vein thrombosis, *SSI* surgical site infection

^a^Mann-Whitney U test

^b^Chi-square test

^c^Only includes acetabular bone grafting

^d^Includes acetabular augmentation

**Fig. 6:**
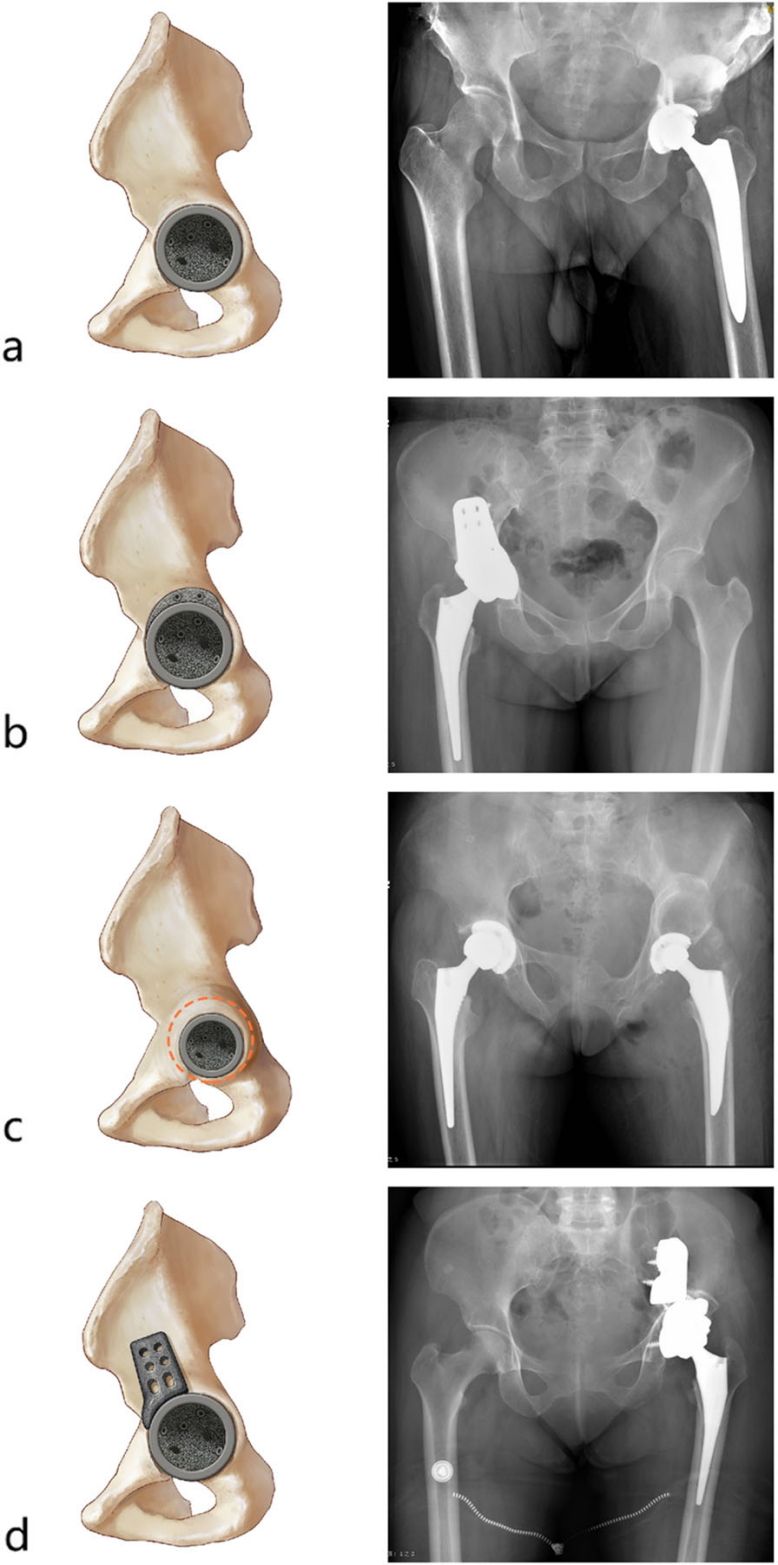
Treatment methods for patients with long-term unreduced hip joint dislocation, secondary osteoarthritis and pseudoarthrosis. **a** Type A places the acetabular component at the position of the original acetabulum (similar to standard total hip arthroplasty). **b** Type B1 places the acetabular component at the position of the original acetabulum, with local structural bone grafting (or augment) on the posterior-superior wall of the acetabulum (similar to total hip arthroplasty in patients with DDH). **c** Type B2 places the acetabular component of small size (commonly 38 mm–42 mm) at the position of the original acetabulum without structural bone grafting (similar to total hip arthroplasty in patients with DDH). **d** Type C places the acetabular component at the position of the original acetabulum, with massive structural bone grafting (or acetabular augmentation). If the original acetabulum cannot be identified during surgery, the acetabular component can be placed at the position of the pseudoacetabulum with a restrictive liner or dual mobility hip system

We also enumerated some recommendations in Table 7 and Fig. 6, which in our opinion might be helpful for surgeons to manage these special patients.

**Table 7:** Recommendations for surgeons to treat patients with long-term unreduced hip joint dislocation, secondary osteoarthritis and pseudoarthrosis

Classification	Acetabular component			Femoral component	Soft-tissue release
	Position	Prosthesis	Bone grafting		
Type A	Original acetabulum	Standard acetabular cup and liner	Commonly unnecessary	Commonly standard femoral stem	Commonly unnecessary or limited soft-tissue release, after removal of the pseudoacetabulum (osteophytes)
Type B1	Original acetabulum	Standard acetabular cup and liner	Structural bone graft (or augmentation)		
Type B2	Original acetabulum	Acetabular cup for DDH (a small-size acetabular cup is commonly chosen)	Commonly unnecessary		
Type C	Original acetabulum (or position of the pseudoacetabulum)	Acetabular component for revision (or placement of the cup in retroversion with application of a restrictive liner)	Structural bone graft (or augmentation)		

*DDH* developmental dysplasia of the hip

### Typical case demonstration

**Fig. 7:**
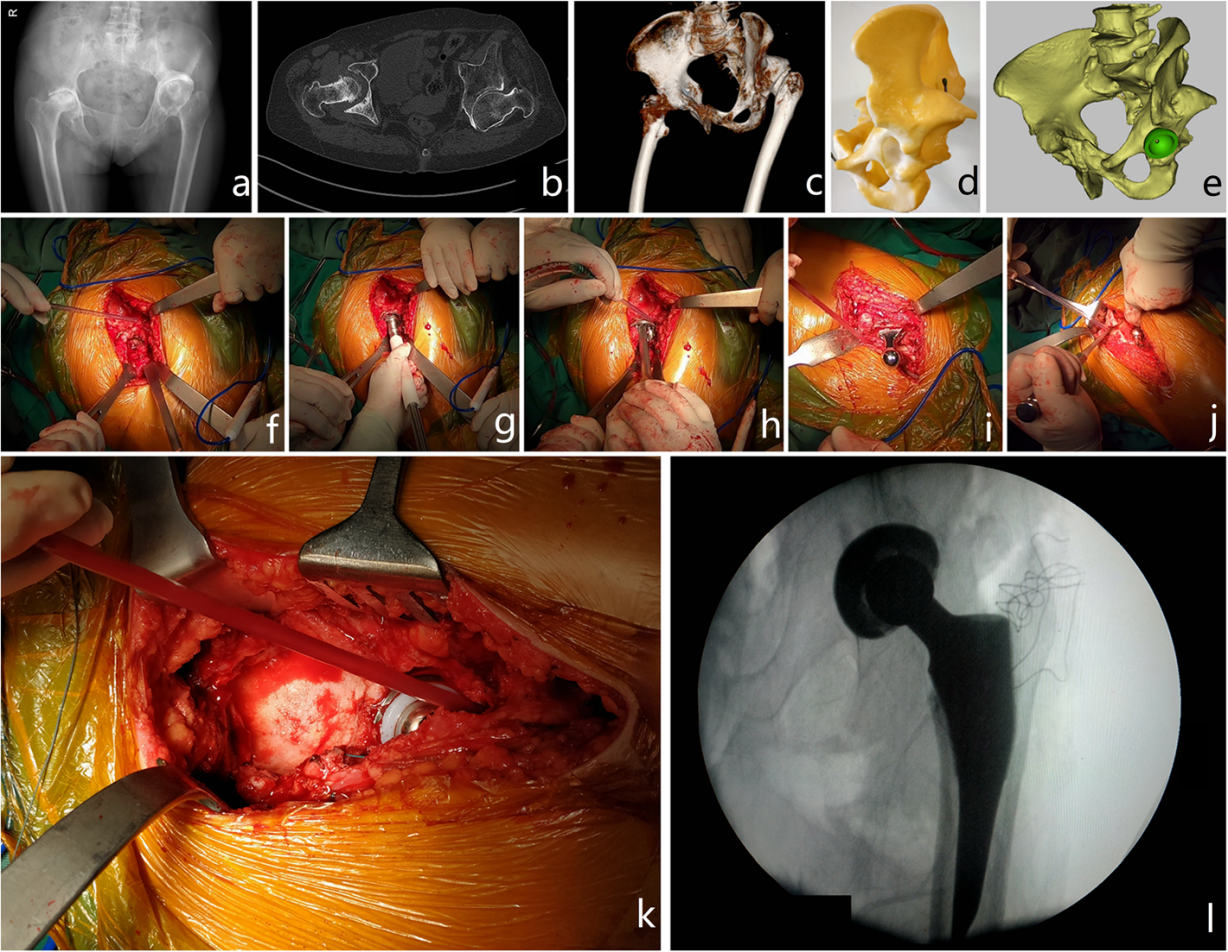
A typical case to show the diagnosis, preoperative evaluation, and surgical procedure of total hip arthroplasty in patients with long-term unreduced hip joint dislocation, secondary osteoarthritis and pseudoarthrosis. This was a 56-year-old female with a complaint of pain in both hips for the past several years. In the most recent 6 months, the patients felt that the aggressive pain increased, especially in the right hip. The pain was described as severe and radiating down the anteromedial thigh regions from the bilateral hips. The patient walked with severe claudication that sometimes necessitated using a cane. She had a history of rheumatoid arthritis and had received systemic steroid treatment. She also reported a previous “pelvic fracture” occurring 23 years ago. **a** Plain radiographs of the pelvis revealed rheumatoid arthritis of the right hip and “osteoarthritis” of the left hip (the aureole sign could be identified). An old pelvic fracture was also identified from the plain radiographs. According to the findings on plain radiographs of the pelvis, the patient was initially diagnosed with “rheumatoid arthritis (right hip) and osteoarthritis (left hip)” and an “old pelvic fracture”. **b** From the axial image of the CT scan, it was identified that the pseudoacetabulum was in retroversion, with subdislocation of the femoral head (the rhombus sign could be identified). **c** On 3D reconstruction of the CT scan, the dislocation of the hip joint was identified, as was the correct position of the original acetabulum. **d** The model of 3D printing clearly showed the pseudoacetabulum. The original acetabulum was also identified. **e** Computer simulation demonstrated that the acetabular component could be implanted on the original acetabulum without bone deficiency. **f** This intraoperative photograph shows the pseudoacetabulum near the original acetabulum, which resembled the Arabic numeral “8”. **g** The original acetabulum was reamed by an acetabular reamer of 40 mm diameter. **h** An acetabular cup was implanted successfully. **i** The femoral component was implanted successfully. **j** The pseudoacetabular wall (osteophyte) was removed; therefore, the muscle tension of the gluteus medius (and gluteus minimus) decreased. The joint prosthesis could be more easily reduced without proximal femoral osteotomy. **k** The joint was reduced successfully. The acetabular cup was well covered without bone deficiency. The pseudoacetabulum could be seen clearly. **l** Intraoperative fluoroscopy showed a good position of the prosthesis. The postoperative radiological examination is shown in Fig. 6c

A typical case of a patient with long-term unreduced hip joint dislocation, secondary osteoarthritis and pseudoarthrosis is shown in Fig. 7. A video is attached to briefly demonstrate the surgical procedure used for this patient.

## Discussion

### Research questions

In this study, there were several predominate research questions (study aims) which may help surgeons in managing patients with long-term unreduced hip joint dislocation, secondary osteoarthritis and pseudoarthrosis, as follows: (1) Evaluation of the diagnostic value for different radiological examination methods. This facilitates identification of the optimized radiological examination for these patients and how to screen these patients on regular X-ray examination. (2) Scientific classification system. Due to the heterogeneity of these patients, a new classification system should be built to help surgeons understand the pathoanatomical nature of the injury. (3) Providing surgical principles for managing these patients. In short, this study primarily focused on addressing the following three questions: how to diagnose, how to classify and how to treat these unique patients.

### Etiology

In this study, all patients had a history of high-energy trauma followed by hip pain and restricted movement. Some of them also had objective evidence to demonstrate hip dislocation even with an acetabular fracture. However, these patients might not be able to receive proper initial medical treatment, resulting in a persistent condition of hip dislocation [6]. Alternatively, in some rare situations, it is suspected that the hip joint might have been initially reduced at the time of injury but that recurrent posttraumatic dislocation of the hip joint also occurred, causing a condition of persistent hip dislocation [12]. According to the radiological features that were identified before arthroplasty and characterized as femoral head dislocation and integral pseudoacetabulum formation, we suspect that in the period from the initial injury to the arthroplasty surgery, the hip joint was continuously in a dislocated condition [9–11]. After the onset of secondary osteoarthritis [18, 19], pain and restricted movement could be identified in these patients.

Another hip disorder that should be included in the differential diagnosis for unreduced hip dislocation is atraumatic hip dislocation [20]. The most common cause of atraumatic hip dislocation is developmental dysplasia [21, 22] (DDH). Generally, leg-length discrepancy, positive Trendelenburg’s sign and limping gait can be found in patients with both traumatic and atraumatic hip dislocation [23, 24]. However, patients with DDH commonly have an insidious onset, chronic progression and long disease course [25]. Meanwhile, patients in this study were characterized as acute onset caused by trauma. Rather than progressive hip pain in patients with DDH [21], serious hip pain could be identified initially in patients with traumatic dislocation, followed by pain remission and re-exacerbation. In addition, patients with mild to moderate DDH rarely have restricted movement of the hip [26]. However, a limited range of motion can be commonly found in patients with unreduced hip dislocation. Demographic characteristics of patients were also different between traumatic and atraumatic hip dislocation (DDH is commonly found in females) [27].

### Radiological diagnosis

If there was a clear history that indicated that the patient had experienced a hip dislocation without a sufficient procedure to reduce the hip joint, the diagnosis might not be difficult with adequate radiological examinations including CT 3D reconstruction of the hip joint. However, in cases of low socioeconomic status, these patients often might not provide a traceable history clearly enough to enable surgeons or radiologists to make the correct diagnosis [6]. In addition, because the pseudoacetabulum forms a barrier at the top of the femoral head, the upward shift distance of the femoral head is commonly not very high. In normal anterior-posterior X-ray examinations, this long-term unreduced hip joint dislocation with pseudoarthrosis resembles “normal” osteoarthritis. In this circumstance, the correct diagnosis becomes crucial. Therefore, the diagnostic value of three different kinds of common radiological examinations were evaluated in this study. The results (Table 2) demonstrated that with 3D reconstruction, CT scans could almost distinguish all these patients from those with other hip disorders (including osteoarthritis, DDH, etc.). In addition, we also believe that, in some patients, 3D printing technology could further help surgeons make the correct diagnosis and identify the original acetabular position, which might be helpful for identifying the implant position of the acetabular component when creating the preoperative plan. Thus, if available, a 3D printing model of the pelvis is recommended for each patient’s preoperative plan. Furthermore, we found two radiological characteristics that might be helpful for screening these patients (Fig. 3). The first is the “aureole” sign, which could be identified on normal anterior-posterior view X-ray examination. It is known that osteoarthritis is characterized mainly by narrowing of the joint space width and by osteophyte formation [28]. In patients with long-term unreduced hip joint dislocation, secondary osteoarthritis and pseudoarthrosis, the femoral head is dislocated to the posterior direction and is sheltered by the anterior bony structures of the hip joint [6]. Meanwhile, the newly formed osteophyte (pseudoacetabulum) surrounds the femoral head. Therefore, in addition to the typical features of osteoarthritis, a semicircular high-density area could be identified around the femoral head in typical cases, named the “aureole” sign. The other radiological sign could be identified on axial CT scans. At the level of the rotation center, the anterior and posterior column and the internal wall of the acetabulum resembles a large letter “I” (in print) in normal people (or patients with osteoarthritis). However, in these patients, due to the upward shift of the rotation center and posterior osteophyte formation, the bony structure looks like a rhombus (at the level of the rotation center). This feature is called the “rhombus” sign. The posterior corner of the rhombus is the newly formed osteophyte (posterior wall of the pseudoacetabulum). We believe that understanding these two special radiological signs might be helpful for surgeons or radiologists to distinguish these patients from “normal” osteoarthritis patients. Note that these two radiological signs are subjective, abstract concepts, and there is no objective quantitative index to describe them. Therefore, we do not include these features in our diagnostic test.

### Radiological measurements

In terms of preoperative radiological evaluation, moderate to integral formation of pseudoacetabulum (Fig. 2c) could be identified in all patients. The time when the pseudoacetabulum was formed remained unknown consequent to the deficiency of the patients’ preoperative longitudinal data. In reports from Nagi [14] and Ilyas [17], which characterized the time period from initial injury to surgery as less than 1 year, no pseudoacetabulum was mentioned. In contrast, according to the report from Pai [6], in four patients with unreduced hip dislocation more than 1 year, a pseudoacetabulum was formed. In our study, with minimally 3 years from initial injury to surgery, all patients had significant pseudoacetabulum formation. Therefore, we hypothesize that the time needed for pseudoacetabulum formation was approximately one to 3 years. The other important issue that we found in the preoperative evaluation was atrophy of the original acetabulum (Fig. 2d). In some patients, the diameter of the original acetabulum was significantly lower than that of the contralateral acetabulum. The reason is not clear, and we have not found related reports. We hypothesize that this phenomenon might be associated with a lack of stress stimulation from noncontact between the original acetabulum and femoral head. Or, in some cases combined with acetabular fracture, the “atrophy” is the outward appearance of bone deficiency of the original acetabulum. These changes might have an influence on the patient classification.

### Classification

To help surgeons and radiologists understand the real pathoanatomical nature of patients with long-term unreduced hip joint dislocation, secondary osteoarthritis and pseudoarthrosis, as well as to make recommendations for surgeons to address this type of patient, we classified the patients into three types (four subtypes). Since in these patients, the femoral head is in a position of posterior-superior dislocation relative to the original acetabulum, a pseudoacetabulum is formed on the posterior-superior direction of the original acetabulum [6]. The patient’s classification and treatment are then determined by the relative positional relationship between the pseudoacetabulum and the original acetabulum. The original acetabular atrophy also influences the classification of patients. In the first case (type A), the original acetabulum retains its original size. Because the distance of the posterior dislocation of the femoral head is relatively far from the original acetabulum and the diameter of the pseudoacetabulum is relatively small, there is no pathological anatomical relationship (contact) between the pseudoacetabulum and the original acetabulum. Therefore, there is no atrophy and no bone deficiency of the original acetabulum (which means the original acetabulum is nearly normal). In the second case (type B1 or B2), the dislocation distance between the femoral head and the original acetabulum is closer, or the diameter of the pseudoacetabulum is larger. At the same time, when the diameter of the original acetabulum maintains its original size, as a result, the anterior-inferior wall (osteophytes) of the pseudoacetabulum invades the posterior-superior part of the original acetabulum. After reaming of the original acetabulum, there is a bone defect in the posterior-superior part of the bone socket (type B1). In the other case, although the anterior-inferior wall of the pseudoacetabulum invades forward, the original acetabulum is atrophied, which helps it “escape” the invasion of the pseudoacetabulum. In this situation, there is commonly no significant bone deficiency on the posterior wall or dome area of the original acetabulum, but the original acetabulum is usually in a small diameter state (type B2). In the last case, the entire original acetabulum is nearly full-filled with the anterior wall of the pseudoacetabulum when the pseudoacetabulum continues to enlarge and to form new osteophytes. The rotation center position of the newly formed hip joint (pseudoarthrosis) will be located at the position of the posterior wall of the original acetabulum. In this situation, after reaming of the original acetabulum, there would be a large bone deficiency in the bone socket of the original acetabulum, or the original acetabulum could not be identified during surgery (type C).

### Surgical treatment

The main purpose of classification is to guide the performance of total hip arthroplasty in these patients (Table 7). For such patients, the treatment methods could be generally divided into two types: hip joint reduction and artificial joint replacement. Since there was a long time (at least 3 years) between the initial injury and the surgery, as well as findings that indicated the onset of hip osteoarthritis, we did not attempt reduction surgeries in these patients [6, 13]. All patients had undergone total hip arthroplasty. There are some uniform characteristics that should be noted during the surgical process of total hip arthroplasty in these patients, as follows: (1) the “pseudo” acetabulum may be mistaken for the original acetabulum, leading to malposition of the acetabular component; (2) the original acetabulum may be atrophied and covered under soft tissue (similar to the situation in DDH patients), making it difficult for surgeons to identify the original acetabulum; (3) bone deficiency may be identified; and (4) the process of reduction may be laborious.

For patients with type A injuries, “standard” surgical procedures could be performed since the original acetabulum remains nearly normal. For patients with type B1 injuries, we expected to identify a bone deficiency in the posterior-superior part of the bone socket after reaming the original acetabulum. In this situation, the “standard” acetabular component could be placed on the original acetabular bone socket with structural bone grafting or augmented to fill the bone deficiency area [25]. Similar to our study, Ilyas [17] has reported that the femoral head might be an optimum selection in this situation. In that study, 13/15 patients with old, short-term hip dislocations (time from injury to surgery equaling less than 1 year) received total hip arthroplasty with the femoral head as a structural bone graft. For patients with type B2 injuries, because the acetabular rim remains integral, an acetabular component with a small diameter (often ≤42 mm) could be placed on the bone socket of the original acetabulum without structural bone grafting. Generally, surgical procedures for implanting the acetabular component for patients with type B injuries are similar to those for patients with developmental dysplasia of the hip [21]. Patients with type C injuries are characterized by severe original acetabular bone deficiency or even a failure to identify the original acetabulum during surgery. We only included one patient with type C injuries in this study. In this case, the acetabular component was placed on the original acetabular position, with multiple augmentations to help fill the severe bone deficiency [29]. Despite the lack of objective evidence, we believe that if the original acetabulum cannot be identified during surgery, the acetabular component could be placed on the bone socket of the pseudoacetabulum [30], while a restrictive liner could be used to avoid potential instability and dislocation of the hip prosthesis resulting from the retroversion of the acetabular component. For patients with type C injury, the surgical procedures for implanting the acetabular component are similar to revision surgery [31] (Fig. 6).

There are another two points to which surgeons should pay additional attention. The first is the selection of a surgical approach. As the direct anterior approach has become an increasingly popular, minimally invasive technique because of decreased pain and accelerated functional recovery, it has become more commonly selected by surgeons for performing total hip arthroplasty in recent years [32]. However, even though three patients underwent surgery via the anterior approach in our study, we do not recommend this technique for these special cases. In such patients, as a consequence of posterior dislocation of the femoral head as well as the blocking of anterior osteophytes of the pseudoacetabulum, the femoral head might be difficult to remove following femoral neck osteotomy when the total hip arthroplasty is performed via the anterior approach. In addition, the walls (osteophytes) of the pseudoacetabulum cannot be well exposed and removed via the anterior approach. Therefore, a posterior approach is recommended for performing total hip arthroplasty in this type of patient. The second point to note is that we recommend that the pseudoacetabulum (osteophytes of secondary osteoarthritis) be completely removed from the surface of the ilium. This is crucial before the process of reducing the prosthetic joint. In fact, although the dislocation distance between the femoral head and original acetabulum is not very far, it is commonly very difficult when surgeons are trying to reduce the hip joint in these patients during surgery. Since the gluteus medius and gluteus minimus are located on the surface of the ilium, when the osteophytes of the pseudoacetabulum are formed, the gluteus medius and gluteus minimus will be pushed away from the surface of the ilium (Fig. 5). This causes the gluteus medius and gluteus minimus to “bypass” the osteophytes, prolonging the distance from the attachment point of the muscle (posterior-superior border of the ilium) to the tip of the trochanter, increasing the muscle tension and causing a failure of reduction. In Ilyas’s study [17], rather than the limited soft-tissue release in our study, they had performed extensive soft-tissue release including the capsule, iliopsoas, iliotibial band, rectus femoris and even hamstrings with another incision. In contrast, in our study, we found that after removing the osteophytes of the pseudoacetabulum, the muscle tension of the gluteus medius and gluteus minimus would be expected to decrease dramatically, making it easy to reduce the joint without over-release of soft-tissue or proximal femoral osteotomy (seen in the video).

### Limitations

There are several limitations of our study, and we have enumerated some major ones. First, the sample size was small. In particular, when the sensitivity, specificity and accuracy were calculated, the insufficient sample size of patients might have resulted in misleading estimates of these indexes (especially the specificity). Second, the follow-up time was relatively short in this study, making us unable to determine the long-term postoperative prognosis and the survival rate of the prostheses. Third, because of the retrospective design of this study, there was a lack of preoperative longitudinal data from the patients. Therefore, we could not identify whether the femoral head was dislocated at the time of initial injury (primary dislocation) or gradually after the injury (secondary dislocation) in some of our patients; furthermore, we could not provide objective evidence to demonstrate how the pseudoarthrosis formed, why there was acetabular atrophy in some patients or what was the relationship between the pseudoarthrosis and the onset of hip pain in the patients. Additionally, when a hip dislocation was identified without emergency reduction, the incidence of avascular necrosis of the femoral head might have been very high. However, our finding could not explain why no significant evidence of avascular necrosis was found in these patients.

## Conclusions

Our study provides detailed information about the radiological diagnosis, preoperative evaluation, classification and surgical treatment for patients with long-term unreduced hip joint dislocation, secondary osteoarthritis and pseudoarthrosis. Patients with this rare injury are difficult to diagnose correctly on standard plain X-ray examinations; therefore, CT scans with 3D reconstruction are strongly recommended when aureole or rhombus signs are identified on patients’ radiological examination results together with a history of hip injuries. According to the morphological characteristics and bone deficiency conditions of the original acetabulum, the patients are classified into three types: For patients with type A injury, which is characterized as the original acetabulum remaining nearly normal, the surgical procedures for total hip arthroplasty are similar to standard total hip arthroplasty. For patients with type B injury, due to atrophy or partial bone deficiency of the original acetabulum, the surgical procedure for total hip arthroplasty might be similar to those for patients with DDH. For patients with type C injury, the situation is similar to that of revision surgery, which is characterized by a larger bone deficiency of the original acetabulum (or the original acetabulum not being found during surgery); therefore, structural bone grafting (or augmentation) might be performed to help reconstruct the acetabular bone socket for prosthetic implantation. By investigating the hip function postoperatively, the prognosis of most patients after total hip arthroplasty is expected to be excellent or good.


**Additional file 3.**


## Supplementary information

**Additional file 1.**

**Additional file 2.**

## Data Availability

All data generated or analyzed during this study are included in this published article.
